# Assessments of Generative Artificial Intelligence as Clinical Decision Support Ought to be Incorporated Into Randomized Controlled Trials of Electronic Alerts for Acute Kidney Injury

**DOI:** 10.1016/j.mcpdig.2024.09.004

**Published:** 2024-10-10

**Authors:** Donal J. Sexton, Conor Judge

**Affiliations:** a Trinity Kidney Centre, School of Medicine, Trinity College Dublin, Dublin, Ireland; b ADAPT Science Foundation Ireland Research Centre, Trinity College Dublin, Dublin, Ireland; c Renal Unit, St James’s Hospital, Dublin, Ireland; d Insight Science Foundation Ireland Research Centre for Data Analytics, Galway, Ireland; e Health Research Board of Ireland Clinical Research Facility, University of Galway, Galway, Ireland

Acute kidney injury (AKI), characterized by an acute deterioration in kidney function, occurs in approximately 25% of hospitalized individuals and is associated with prolonged stay, higher cost, and increased morbidity and mortality.^1^ Clinical interventions that prevent or reduce AKI severity include altering medication and contrast administration, treating underlying sepsis, and optimizing blood pressure.^1^Conceptually, early identification of AKI through electronic alerts (e-alerts) may offer an opportunity to alter its course and improve patient outcomes; however, studies to date have failed to demonstrate consistent benefit. Frameworks of action in response to an e-alert capable of reproducibly altering the clinical course of AKI are required.

Although studies to date have focused on simpler models, clinical decision support tools (CDSTs) based on generative artificial intelligence (AI) are likely to predominate in future given their potential for superior clinical reasoning while aligning with physician clinical workflows.^2^ Generative AI refers to AI models, such as large language models, trained on extensive medical data sets to perform complex language tasks and generate human-like responses.^3^ These models, including ChatGPT (generative pre-trained transformer), Llama, Med-PaLM, and BioMedLM, are designed to assist in clinical decision making by processing vast amounts of health care information.^3^ Contemporary generative AI models are becoming increasingly adept at multimodal data incorporation, a feature that could harness the potential of this technology to improve clinical prediction and management of AKI, for example, taking as input unstructured electronic health record (EHR) notes and existing medication ordersand providing specific advice to cease medications impeding renal autoregulation.^2^^,^^4^ Generative AI models could be developed as CDSTs in AKI by training on medical question type format for the optimal subsequent management choice at each step based on kidney function and known risk factors for AKI incidence and worsening AKI in addition to clinical guidelines for AKI such as Kidney Disease: Improving Global Outcomes.^3^ Training would likely entail fine-tuning an large language models (eg, Llama 3.1 and Mistral 7B) on extensive AKI data sets ensuring that CDST recommendations align with Kidney Disease: Improving Global Outcomes clinical practice guidelines and local policies.

Less experienced members of the clinical team, with no specific nephrology training, may be the first to interact with the e-alert, and a lack of direct involvement by a nephrologist after the activation of an e-alert has been cited as a potential explanation for the lack of observed benefit in randomized trials. However, availability of such resources and variability in expert opinion will limit the scalability, global generalizability, and standardization of this approach. Perhaps, the development of generative AI models providing reproducible expert recommendations will improve patient outcomes in these urgent care issues, particularly in resource-limited countries where nephrology input may be more limited.

Investigators have highlighted limitations of contemporary generative AI models in more specialist medical subdomains, which may require more nuance, for example, glomerular disease, where ChatGPT found poor accuracy and limited repeatability.^5^ This limitation may be overcome by additional domain-specific model training or retrieval augmented generation; however, in specialized topics, training material, particularly open access sources will be more limited.

Google DeepMind provides the state-of-the-art prediction for AKI in the form of a rolling updated probability of occurrence in the subsequent 48 hours.^6^ Widespread implementation of this model into clinical practice has not been followed,^6^ and randomized controlled trials of e-alerts to date have not found clear consistent benefits in the management of patients with AKI.^7^ These studies comprise a wide variety of designs, including parallel group designs (both single-center and multicenter), double-blind and open-label formats, and stepped-wedge cluster designs. This diversity in the study design reflects the complexity and challenges of evaluating e-alert systems in clinical settings. Each study had potential benefits and limitations, with various degrees of blinding of clinicians, investigators, and data analysts, and challenges such as unblinding, loss of allocation concealment, contamination, carryover of learned behaviors, as well as alert fatigue. Many studies also randomized at the point of AKI occurrence rather than at hospitalization, which may limit the effectiveness of the alert because earlier intervention might actually prevent the onset of AKI rather than just managing it after the fact.

Although the use of commercial EHR alerts in these studies facilitates work flow and generalizability, the underlying assumptions and performance of these models have not been rigorously tested, as was recently highlighted for the EPIC AKI e-alert.^8^ Lack of impact on clinical outcomes may also be contributed to by the fact that physicians may already be aware of the AKI, and therefore, their behavior is unaltered by the alert. Debate also exists as to whether improved outcomes could be elicited by restricting alerts to more severe derangements in kidney function or to those more likely to benefit rather than blanket alerts for all AKIs.

Although much of the focus of the research community is on building accurate prediction models, the actual implementation of AI models into clinical practice has many more facets to consider and requires fastidious oversight and ongoing surveillance.^9^ de Hond et al^9^ have proposed 6 phases of AI prediction model implementation: (1) data preparation; (2) model development; (3) model validation; (4) software development; (5) model impact assessment; and (6) model implementation into daily health care practice. Unfortunately, literature pertaining to the later phases of this process is more sparse.^9^ At an institutional level, there is a requirement to develop procedures around assessments of transparency of the modeling process, external validation, human-AI interaction, software security, software testing, risk management, AI model maintenance and updating, education, monitoring, and auditing.^9^ In addition, the creation of seamless infrastructure to allow the embedding of software within EHRs requires robust data management and security standards and the ability to process real-time data and deal with system failures or downtime.^9^ Auditing of AI includes ongoing assessment of model performance in addition to detecting alterations in input variables that could lead to issues such as data set shift, for example, in AKI, a change in estimated glomerular filtration rate equation or creatinine assay by the local laboratory could have implications for model performance if these differ from those used in the model-derivation cohort.

Alert fatigue among clinical teams could reduce the effectiveness of AI-based tools, overcoming such issues requires a combination of optimizing human-computer interaction methodology in addition to comprehensive education and training on the benefits of AI models and the interpretation of their outputs.^10^ Health care professional education and familiarization with AI is of paramount importance to its successful implementation into clinical practice. Our hypothetical randomized controlled trial design protocol incorporates an assigned period of AKI model–specific education of health care workers at each site before randomization (Figure). This period is envisaged to constitute educational sessions to nurses and doctors on how the model works and the implications of the CDST recommendations which form the output. These educational sessions need to be designed using robust human factors methodology like user centered design and formal usability tests. In a broader sense, each health care institution will be required to develop or adopt accredited educational programs focused on raising awareness of the benefits and limitations of AI in medicine. In the UK National Health Service, an initiative known as the AI Lab is involved in raising AI literacy among health care professionals through webinars, workshops, and academic collaborations to develop curricula aimed at equipping National Health Service staff to work alongside AI.^11^

**Figure:**
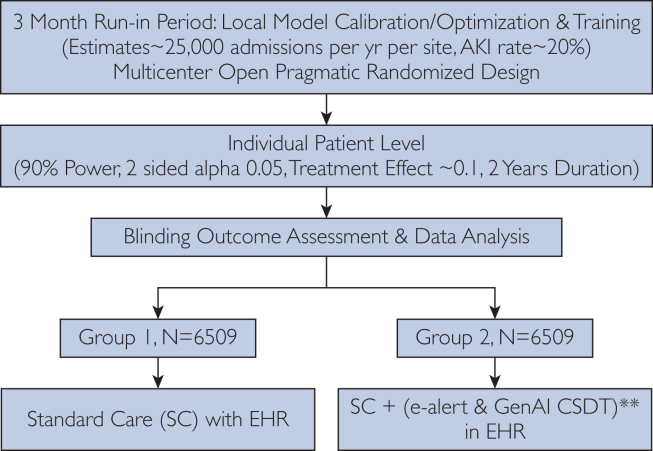
Hypothetical protocol design of a randomized controlled trial of an acute kidney injury (AKI) electronic alert and a generative artificial intelligence (AI) model–based Clinical Decision Support tool (CDST). The primary outcome is a composite of the severity of AKI as defined by Kidney Disease: Improving Global Outcomes (grades 1-3) and duration of dialysis (in days). The sample size is calculated to reduce the primary composite outcome by 10%. Sample size per group is calculated using the Z-test for 2 proportions formula. Secondary outcomes are proportion admitted to the intensive care unit and kidney function at the 5-year follow-up (estimated glomerular filtration rate, urinary albumin to creatinine ratio, and chronic kidney disease stage). Mortality is not part of the primary or secondary outcomes as it may be unreasonable to expect an AKI CDST to have a large effect on mortality. Health care providers are not blinded, but the research team and statisticians are blinded to intervention assignment. ∗∗In the active intervention group, both the AKI prediction e-alert model and CDST are housed within 1 GUI embedded in the electronic health record interface. In future, both the AKI prediction model and the CDST management may be encompassed solely within the interactive generative AI GUI, which could also then be embedded into the electronic patient record. However, at the time of writing, Google DeepMind is the state-of-the-art prediction model, which is not based on generative AI. In this study, an e-alert generated by DeepMind AKI algorithm is followed by a generative AI CDST for AKI. EHR, electronic health record.

The implications of bias in AI ought to be considered and deliberately assessed for throughout the clinical implementation process because it can lead to inequitable health care outcomes.^3^^,^^12^ Common forms of bias in AI include underrepresentation of subpopulations in the data used to train the model, which can result in dipropionate benefits or disadvantages for certain patients.^12^ A notable example of a bias in a widely used algorithm, potentially affecting millions of individuals was reported by Obermeyer et al^13^ in which Black patients were underrepresented in the model development stage owing to systematic bias in health care access but were thus inaccurately considered lower risk by the model.^13^ Model development of diverse data set as representative as possible of the target population, deliberate oversampling of minority subgroups and clear acknowledgment of model limitations in certain subgroups are important methods to limit bias in AI.^12^ Analogous to model performance and data set shift, the new development of bias in the AI model must also be continually audited, monitored for, and addressed in the clinical implementation phase of AI. Periodic employment of bias detection methodology such as fairness audits is also needed on an ongoing basis.

Transparency, accountability, and explainability as they relate to AI have considerable implications for its adoption in clinical medicine.^3^ Transparency refers to clarity and openness on model operations, outputs and recommendations, and their communication to stakehodlers.^3^^,^^14^ AKI e-alerts and CDST based on AI must be transparent to clinicians about the model development process, how the algorithm works, and the limitations of the model. In order for clinical teams to implement an AI-based model CDST, they must understand how the model reaches conclusions and recommendations and each clinician must decide what implications the model will have on their individual clinical practice and how they might feel about model recommendations.^3^^,^^14^

Accountability in relation to AI in medicine refers to the attribution of responsibility for a course of action influenced by the output of the AI algorithm, and if harm ensures, having processes in place to discern where the responsibility lay, the model development, the software, or the clinical interpretation of the model output.^15^ The establishment of local robust governance structures defining accountability at each stage in the implementation is essential and will require tuning in different jurisdictions to ensure cognizance of local AI legislation.^15^

Explainability refers to the ability of the AI model to provide interpretable detailed descriptions on how the model decided on a certain predictions or recommendations.^16^^,^^17^ Concerns over explainability represent 1 of the major limitations to the widespread adoption of AI in medicine, particularly for models that provide no explanation or so called “Black Box” recommendations, despite demonstration of excellent performance in simulated clinical environments.^3^^,^^16^^,^^17^ These concerns have led to an emphasis on increased AI model explainability; however, although explainability is an inherently crucial element, there are limitations to explanations provided by model diagnostics and a degree of error around explainability metrics must be anticipated.^16^^,^^17^

Many factors influence whether e-alerts and CDSTs ultimately lead to improved patient care including efforts at supporting physicians in their decision making and clinical workflows.^18^ Multimodal AI may represent an opportunity for optimal human-computer interaction with potential for interactive visualization, EHR embedding, integration, and user centered design.^4^ Nonetheless, assessing the effectiveness of such complex interventions ought to be subjected to randomized assessment of benefit, safety, and performance in terms of repeatability and standardization of the recommended approach. These trials need to be preregistered^19^ and reported in a standardized fashion.^20^ Ultimately, the long-term success of AI in health care will rely on multicenter trials and external validation to ensure generalizability across diverse health care settings and patient populations. Figure demonstrates a hypothetical protocol for an open pragmatic parallel randomized trial design for such an assessment of generative AI as a CDST for AKI after local model calibration, the importance of which was recently highlighted by Cao et al^1^ in relation to the DeepMind AKI algorithm.

## Potential Competing Interests

Dr Sexton is funded by the Health Research Board of Ireland, grant number HRB-ARPP-P-011 (completed in 2022); reports honoraria for presentations from Astrazeneca, Ireland, Boehringer Ingelheim, Ireland, and Takeda, Ireland; travel sponsorship to the British Transplant Society meeting 2024 from Takeda; and participation in the advisory board meeting for Astrazeneca, Boehringer Ingelheim, and Takeda; and is the Honorary Secretary of the Irish Nephrology Society. Dr Judge is funded by the Health Research Board of Ireland Postdoctoral Clinician Scientist Fellowship 2023, grant number CSF-2023-016; reports participation in the data safety monitoring board of Salt supplementation in older adults with orthostatic intolerance disorders (STOOD): a phase IIa randomised controlled trial of sodium supplementation in those consuming moderate salt intake—Health Research Board; and is the Secretary of Irish Nephrology Society Research and Audit Committee.
